# Is preoperative protein-rich nutrition effective on postoperative outcome in non-small cell lung cancer surgery? A prospective randomized study

**DOI:** 10.1186/s13019-016-0407-1

**Published:** 2016-01-19

**Authors:** Seyda Ors Kaya, Tevfik Ilker Akcam, Kenan Can Ceylan, Ozgur Samancılar, Ozgur Ozturk, Ozan Usluer

**Affiliations:** Department of Thoracic Surgery, Dr. Suat Seren Chest Diseases and Thoracic Surgery Training and Research Hospital, Izmir, Turkey

**Keywords:** Albumin, Lung cancer, Preoperative-nutrition

## Abstract

**Objective:**

Protein-rich nutrition is necessary for wound healing after surgery. In this study, the benefit of preoperative nutritional support was investigated for non-small cell lung cancer patients who underwent anatomic resection.

**Methods:**

A prospective study was planned with the approval of our institutional review board. Fifty-eight patients who underwent anatomic resection in our department between January 2014 and December 2014 were randomized. Thirty-one patients were applied a preoperative nutrition program with immune modulating formulae (enriched with arginine, omega-3 fatty acids and nucleotides) for ten days. There were 27 patients in the control group who were fed with only normal diet. Patients who were malnourished, diabetic or who had undergone bronchoplastic procedures or neoadjuvant therapy were excluded from the study. Patients’ baseline serum albumin levels, defined as the serum albumin level before any nutrition program, and the serum albumin levels on the postoperative third day were calculated and recorded with the other data.

**Results:**

Anatomic resection was performed by thoracotomy in 20 patients, and 11 patients were operated by videothoracoscopy in the nutrition program group. On the other hand 16 patients were operated by thoracotomy and 11 patients were operated by videothoracoscopy in the control group. In the control group, the patients’ albumin levels decreased to 25.71 % of the baseline on the postoperative third day, but this reduction was only 14.69 % for nutrition program group patients and the difference was statistically significant (*p* < 0.001). Complications developed in 12 patients (44.4 %) in the control group compared to 6 patients in the nutrition group (*p* = 0.049). The mean chest tube drainage time was 6 (1–42) days in the control group against 4 (2–15) days for the nutrition program group (*p* = 0.019).

**Conclusions:**

Our study showed that preoperative nutrition is beneficial in decreasing the complications and chest tube removal time in non-small cell lung cancer patients that were applied anatomic resection with a reduction of 25 % in the postoperative albumin levels of non-malnourished patients who underwent resection.

## Background

Cancer surgery and treatment is a process that poses a severe burden to the body and increases catabolism, thereby increasing the patient’s protein and energy needs [1–4]. On the other hand, normal levels of protein and energy accelerate the recovery process. This has a definite association with nutrition [3, 4]. This also applies to patients with lung cancer and has been investigated [1, 5, 6]. However, most of these studies associate preoperative nutrition status with prognosis. [1, 5–8]. In the present study, we aimed to investigate the effect of surgery on the serum albumin levels during the early postoperative period as well as the association between preoperative nutrition status and prognosis. Based on this, we compared the early postoperative data of the patients who had been given preoperative nutritional support with that of the other patients who all underwent anatomic lung resection due to non-small cell lung cancer [NSCLC].

## Methods

Among the patients who were admitted to our clinic between January 2014 and January 2015 those who were operated due to non-small cell lung carcinoma were evaluated. For the purposes of our study, complete randomization was the chosen randomization method and randomness was achieved by classifying patients according to the last digits of their protocol numbers where patients with odd numbers were included in the control group, whereas patients with even numbers were included in the nutrition group. The G*Power software was used for the Post Hoc Power analysis and the power ratio calculated using albumin as the valuable was found as 99.9 %. All patients gave written informed consent and approval was obtained from the local Ethics Committee for the study, which was designed as a prospective study (No:238).

The albumin levels of the included patients were measured at baseline and on the postoperative 3^rd^ day. The body mass indices (BMIs) were calculated. The BMI classification recommended by the World Health Organization (WHO) was used. 31 of the randomized patients were applied a preoperative nutrition program with immune modulating formulae (enriched with arginine, omega-3 fatty acids and nucleotides) for ten days. 27 patients in the control group were given normal diet without any additional nutritional products. Care was taken to distribute the groups homogeneously. Patients who were malnourished (BMI less than 18.5) and patients with metabolic disorders were excluded from the study to form a homogenous group and ensure that the patients’ nutritional level is not affected by any factor other than the operation. In addition, patients, who had received pre-operational radiotherapy and/or chemotherapy, patients who were under parenteral nutritional support, patients with chronic renal and hepatic disorders, those who had another operation or major trauma recently or received blood transfusion recently, patients with coagulation disorders and abnormal laboratory findings were excluded. Also, in order to homogenize the operation effect, patients with previous history of extended and/or bronchoplastic resection were also excluded. Tube thoracostomy was discontinued in patients with discontinued air drainage and a daily fluid drainage ≤ 200 cc. Patients with an air drainage lasting ≥ 7 days were considered to have prolonged air leakage The patients’ ages, genders, preoperative FEV_1_ values, presence of concomitant diseases, histology results, pathological stages, operation types, postoperative complications and chest tube drainage times as well as their albumin and BMI values were recorded.

### Statistical analysis

Data were analysed using the SPSS 22.0 (IBM statistics for Windows version 22, IBM Corporation, Armonk, New York, United States) and PAST (Hammer, Ø., Harper, D.A.T., Ryan, P.D. 2001. Paleontological Statistics) software packages. The normality of the univariate data was assessed using the Shapiro-Wilk test, while the variability coefficient and the multivariate normality were tested through the Mardia, Doornik & Omnibus test. In the comparison of two independent groups, the Independent T-Test was applied together with the bootstrap results, while the Mann–Whitney U-test was employed using the Monte Carlo simulation technique. The interaction of repeated measures of dependent variables was observed based on the General Linear Model-Repeated Anova (Wilks’ Lambda) test. Categorical data were compared through Pearson’s Chi-Square test with the Monte Carlo simulation technique. Quantitative data were expressed in mean ± SD (standard deviation) and the median range (maximum-minimum) values. Categorical data were expressed in n (number) and percentage (%). Data were evaluated with a 95 % confidence interval and statistical significance was based on a value of *p* < 0.05.

## Results

In this prospective study, 54 male (93.1 %) and 4 female (6.9 %) patients were included. The mean ages of the nutrition group (Group1 - G1) and the control group (Group 2 - G2) were 57.80 ± 9.70 (37–74) and 59.04 ± 7.61 (47–72) respectively. The body mass indices (BMIs) were 25.2 ± 4.13 in Group 1 and 26.7 ± 3.49 in Group 2. No significant difference was found when these variables were statistically evaluated. In addition, the mean BMIs in patients who underwent thoracotomy and in those who underwent VATS were 25.21 ± 4.11 and 26.92 ± 3.31 respectively (*p* = 0.104). 13 (41.9 %) of the patients in the study group (G1) had concomitant diseases. Their distribution was as follows: Five patients had hypertension, 3 had chronic obstructive pulmonary disease (COPD), 2 had coronary artery disease, 1 had multi-nodular goiter, 1 had congestive heart failure and 1 had asthma. 9 (33.3 %) of the patients in G2 concomitant diseases. Their distribution was as follows: 4 had hypertension, 2 multi-nodular goiter, 1 had ankylosing spondylitis, 1 had parkinson’s disease and 1 had peripheral vascular disease. No concomitant diseases were reported in the other patients. The calculated mean FEV_1_ values were 71.55 % in G1 and 74.50 % in G2 (Table 1). The mean baseline albumin values in the nutrition and control groups were comparable (4.15 ± 0.27 and 4.20 ± 0.43 respectively). The statistical analysis showed a homogenous distribution in all these demographical data.

**Table 1:** Patients’ demographic data

		Control group	Nutrition group	Total	*P* value
Age^a^		59.04 ± 7.61/year	57.80 ± 9.70/year	58.35 ± 8.8/year	0.482
Sex	Male	25 (92.6 %)	29 (93.5 %)	54 (93.1 %)	1
	Female	2 (7.4 %)	2 (6.5 %)	4(6.9 %)	
FEV-1^a^		74.5 ± 15.01 %	71.55 ± 15.52 %	72.84 ± 15.23 %	0.594
BMI	Thoracotomy	26.7 ± 4.20	24.0 ± 3.69	25.21 ± 4.11	0.051
	VATS	26.6 ± 2.26	27.3 ± 4.19	26.92 ± 3.31	0.632
	Total	26.7 ± 3.49	25.2 ± 4.13	25.86 ± 3.88	0.138
Operation Type	Thoracotomy	16 (59.3 %)	20 (64.5 %)	36 (62.06 %)	0.788
	VATS	11 (40.7 %)	11 (35.5 %)	22 (37.94 %)	
Resection Type	LUL	5 (18.52 %)	9 (29.03 %)	14 (24.14 %)	0.788
	LLL	4 (14.81 %)	6 (19.35 %)	10 (17.24 %)	
	RLL	5 (18.52 %)	6 (19.35 %)	11 (18.97 %)	
	L-P	2 (7.41 %)	5 (16.13 %)	7 (12.07 %)	
	RUL	8 (29.63 %)	3 (9.68 %)	11 (18.97 %)	
	RSBL	2 (7.41 %)	2 (6.45 %)	4 (6.89 %)	
	RIBL	1 (3.7 %)		1 (1.72 %)	
Drainage Time^b^		6 (42–1)/day	4 (15–2)/day	4.9 ± 2.26/day	0.019
Complication	PAL	7 (58.33 %)	4 (66.67 %)	11 (18 %)	
	Atelectasis	3 (25 %)	1 (16.67 %)	4 (6.8 %)	
	Pneumonia	1 (8.33 %)	1 (16.67 %)	2 (3.4 %)	
	Arhytmia	1 (8.33 %)		1 (1.7 %)	
	Total	12 (44.4 %)	6 (19.4 %)	18 (31.04 %)	0.049
	AC	17 (62.96 %)	12 (38.71 %)	27 (46.55 %)	0.122
Histology	SCC	9 (33.33 %)	18 (58.06 %)	27 (46.55 %)	
	Carcinoid	1 (3.7 %)	1 (3.23 %)	2 (6.9 %)	
Stage	IA	7 (25.93 %)	11 (35.48 %)	18 (31.04 %)	0.608
	IB	6 (22.22 %)	3 (9.68 %)	9 (15.52 %)	
	IIA	8 (29.63 %)	7 (22.58 %)	15 (25.86 %)	
	IIB	3 (11.11 %)	6 (19.35 %)	9 (15.52 %)	
	IIIA	3 (11.11 %)	4 (12.9 %)	7 (12.06 %)	
Concomitant Diseases	Hypertension	4 (44.44 %)	5 (38.46 %)	9 (40.91 %)	
	COPD		3 (23.08 %)	3 (13.64 %)	
	CAD		2 (15.38 %)	2 (9.09 %)	
	MNG	2 (22.22 %)	1 (7.69 %)	3 (13.64 %)	
	CHF		1 (7.69 %)	1 (4.55 %)	
	Asthma		1 (7.69 %)	1 (4.55 %)	
	AS	1 (11.11 %)		1 (4.55 %)	
	Parkinson	1 (11.11 %)		1 (4.55 %)	
	PVD	1 (11.11 %)		1 (4.55 %)	
	Total	9 (33.3 %)	13 (41.9 %)	22 (37.9 %)	0.592

Pearson Chi-Square Test (Monte Carlo) - Mann Whitney U Test (Monte Carlo) - Independent T Test (Bootstrap)

^a^Mean ± SD, ^b^Medyan Range (Maximum- Minimum), n (%)

BMI: Body Mass Index; VATS: Video-assisted thoracoscopic surgery; LUL: Left Upper Lobectomy; LLL: Left Lower Lobectomy; RLL: Right Lower Lobectomy; L-P: Left Pneumonectomy; RUL: Right Upper Lobectomy; RSBL: Right Superior Bilobectomy; RIBL: Right İnferior Bilobectomy; PAL: Prolonged Air Leak; AC: Adenocarcinoma; SCC: Squamous Cell Carcinoma; CAD: Coronary Artery Disease; COPD: Chronic obstructive pulmonary disease; MNG: Multinodular goiter; CHF: Congestive heart failure; AS: Ankylosing spondylitis; PVD: Peripheral vessel disease

Of the 31 patients in Group 1, 20 (64.5 %) underwent thoracotomy and 11 (35.5 %) underwent videothoracoscopic lung resection. The control group had a similar distribution with 16 (59.3 %) patients who underwent thoracotomy and 11 (40.7 %) VATS (*p* = 0.788). All patients underwent anatomic resection, and the distribution in the nutrition group was as follows: 9 (29.03 %) patients had left upper lobectomy (LUL), 6 (19.35 %) had left lower lobectomy (LLL), 6 (19.35 %) had right lower lobectomy (RLL), 5 (16.13 %) had left pneumonectomy, 3 (9.68 %) had right upper lobectomy (RUL), 2 (6.45 %) had right superior bilobectomy (RSBL). The distribution in the control group was: 8 (29.63 %) RUL, 5 (18.52 %) LUL, 5 (18.52 %) RLL, 4 (14.81 %) LLL, 2 (7.41 %) RSBL, 2 (7.41 %) left pneumonectomy, 1 (3.7 %) right inferior bilobectomy (RIBL). The postoperative pathology reports of the patients in G1 revealed squamous cell carcinoma in 18 (58.06 %) patients, adenocarcinoma in 12 (38.71 %) and carcinoid tumor in 1 (3.23 %). The distribution in G2 was: adenocarcinoma in 17 (62.96 %) patients, squamous cell carcinoma in 9 (33.3 %) and carcinoid tumor in 1 (3.7 %). The stages of the patients were as follows: 11 (35.48 %) patients were stage 1A, 3 (9.68 %) were stage 1B, 7 (22.58 %) were stage 2A, 6 (19.35 %) were stage 2B; 4 (12.9 %) were stage 3A in the nutrition group, and 7 (25.93 %) were stage 1A, 6 (22.22 %) were stage 1B, 8 (29.63 %) were stage 2A, 3 (11.11 %) were stage 2B and 3 (11.11 %) were stage 3A in the control group.

**Fig. 1:**
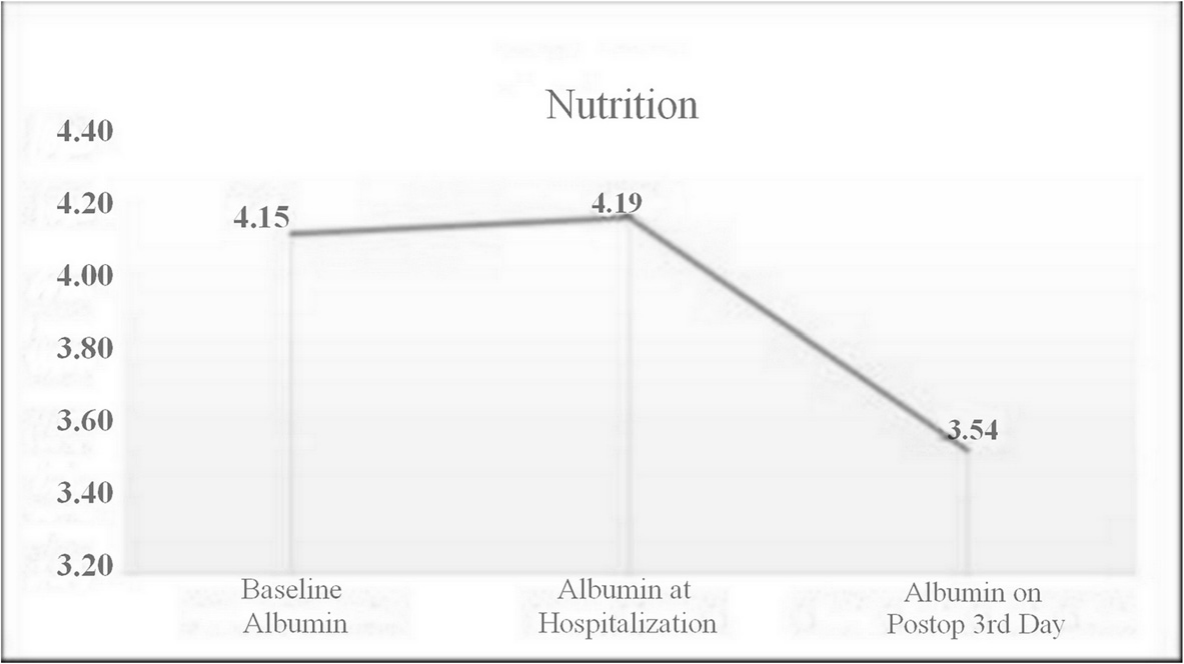
Nutrition group albumin level

In the light of all these data, postoperative follow-ups of the patients were conducted and their detailed values were noted. In the control group, while the mean baseline albumin value was 4.20 ± 0.43 mg/dl, the mean value on the third postoperative day was 3.12 ± 0.35 mg/dl. A reduction of %25.71 in the albumin levels was observed. In the nutrition group, while the mean baseline albumin value was 4.15 ± 0.27 mg/dl, the mean value on the third postoperative day was 3.54 ± 0.35 mg/dl (reduced by 14.69 %) (Fig. 1). The difference of the reduction rates was statistically significant (*p* <0.001). With respect to thoracotomy and VATS, the change in albumin levels of the patients who underwent thoracotomy was 0.58 ± 0.33 in G1 and 1.04 ± 0.31 in G2 (*p* < 0.001). The change in albumin levels of the patients who underwent VATS was 0.65 ± 0.27 in G1 and 1.15 ± 0.44 in G2 (*p* = 0.005) (Table 2).

**Table 2:** Patients’ albumin levels

	Preoperative albumin level (mg/dl)	Postoperative 3.day albumin level (mg/dl)	Albumin change	*P* value
C.G. VATS	4,30 ± 0,45	3,15 ± 0,41	1,15 ± 0,44 (26.74 %)	0,005
N.G. VATS	4,25 ± 0,23	3,60 ± 0,35	0,65 ± 0,27 (15.29 %)	
C.G. Thoracotomy	4,13 ± 0,42	3,09 ± 0,31	1,04 ± 0,31 (25.18 %)	<0,001
N.G. Thoracotomy	4,09 ± 0,28	3,51 ± 0,36	0,58 ± 0,33 (14.18 %)	
C.G. Total	4,20 ± 0,43	3,12 ± 0,35	1,08 ± 0,37 (25.71 %)	<0.001
N.G. Total	4,15 ± 0,27	3,54 ± 0,35	0,60 ± 0,31 (14,69 %)	

General Linear Model Repeated Anova (Wilks’ Lambda)

Mean ± Standard deviation

*C.G* Control group, *N.G* Nutrition group, *VATS* Video-assisted thoracoscopic surgery

**Fig. 2:**
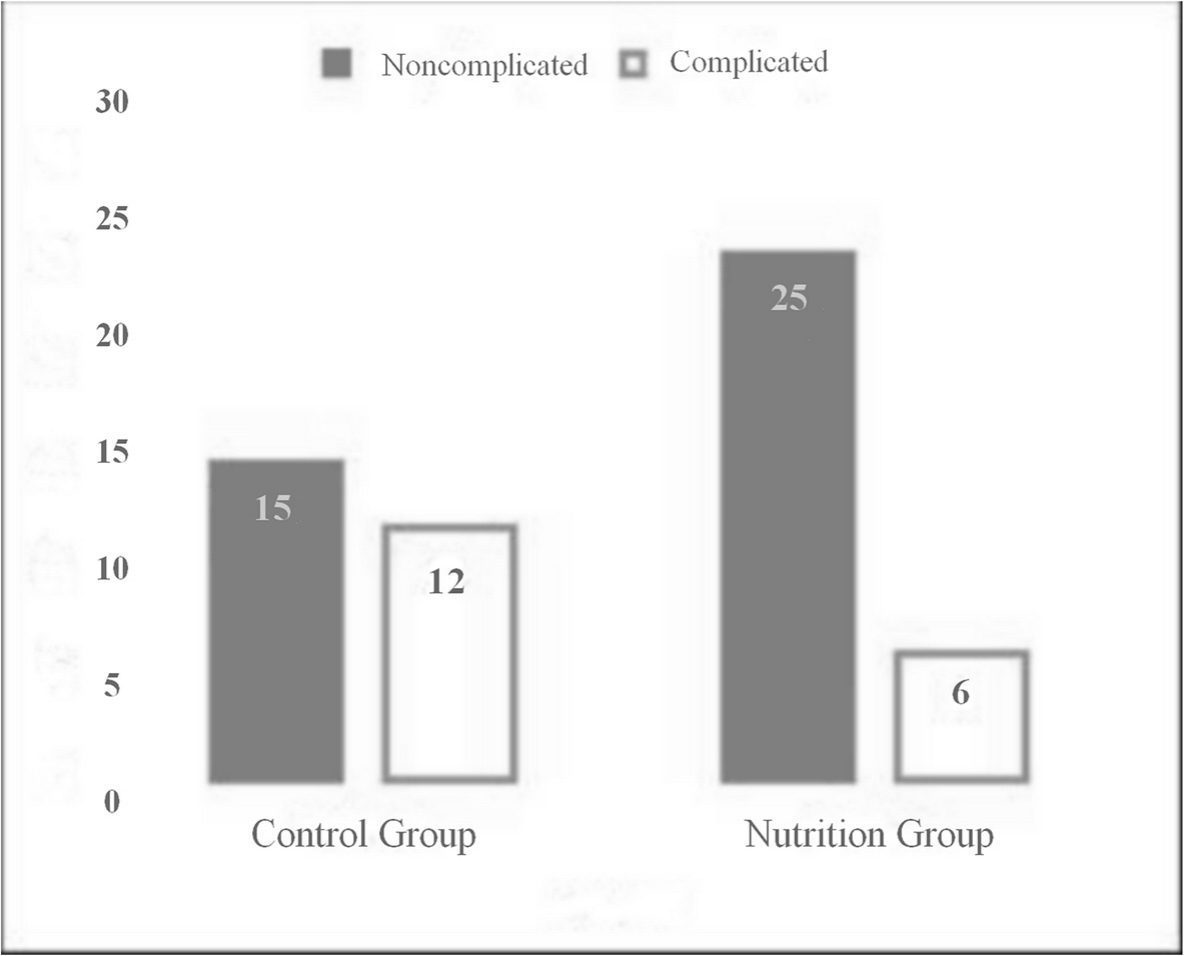
Patients’ postoperative complications graphic

**Fig. 3:**
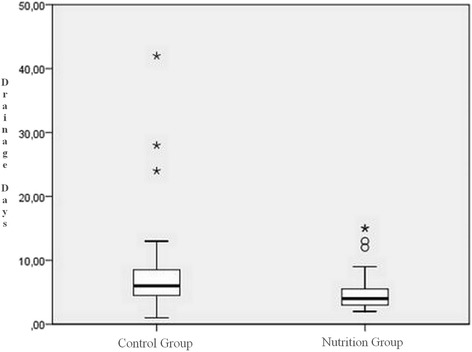
Drainage days graphic

This change in albumin levels was also reflected in the complications and drainage times. Of the 27 patients in the control group, 12 (44.4 %) developed complications. Of these 12 patients, 7 (58.33 %) had prolonged air leak, 3 (25.0 %) had atelectasis requiring bronchoscopy, 1 (8.33 %) had pneumonia and 1 (8.33 %) had cardiac arhythmia. On the other hand, in G1, of the 31 patients only 6 (19.4 %) developed complications; 4 (66.67 %) had prolonged air leak, 1 (16.67 %) had atelectasis requiring bronchoscopy and 1 (16.67 %) had pneumonia (Fig. 2). There was a statistical difference between the two groups with respect to complication development rates (*p* = 0.049). The mean tube drainage times were 6 (42–1)/day in the control group and 4 (15–2)/day in the nutrition group (*p* = 0.019) (Fig. 3).

## Discussion

Nutritional arrangements required in lung cancer have not become clear yet [1, 5, 6, 8]. In cancer surgery and treatment, which is a process posing a severe catabolic burden, the form, timing and extent of nutritional support is very important. Considering that especially malnutrition is a very important factor affecting mortality and morbidity during the postoperative period and can be seen in 20-50 % of patients postoperatively, this becomes even more important [9]. The studies conducted on nutrition in lung cancer surgery are related mainly to the reflections of the preoperative nutritional levels in the postoperative status [1, 3, 5–7, 10, 11]. When we reviewed these studies in detail, we found the investigation on a large population of patients by French Surgeons. In the mentioned study, the nutritional statuses of the patients were examined at four levels by their BMIs, namely underweight, normal, overweight and obese, and it was concluded that the operative mortality was low in the normal BMI, overweight and obese patient groups, but higher in the underweight group was higher. Moreover, the complication rate was significantly higher in the underweight group compared to the normal group [5]. In another study on preoperative nutritional levels, calculations of body mass index, triceps diameter and subscapular skin fold were made, and higher postoperative protein-energy malnutrition was found in all patients with mortality compared to the other patients [7]. A similar study investigated the postoperative mortality and morbidity statuses of patients operated due to lung cancer and showed that the preoperative nutritional levels, pulmonary functions and maximum expiratory pressures were lower in the patients who died or required re-ventilation in the postoperative period [6]. In another patient group aged older than 70 years who underwent operation due to lung cancer, the life time of the malnourished patients with BMI values below 18.5 was lower than the other group. This study also suggested that such malnourished patients should be given nutritional support both in the preoperative and postoperative period [10]. This was also concluded in other similar studies, and a rehabilitation program including nutritional support was suggested to reduce the rates of various postoperative complications [1, 5, 8–10].

Although there are a few studies reporting that low nutritional levels is not associated with complications [12–14], all these studies show that nutritional status has direct effects on the postoperative complications of lung cancer surgery. Our study was planned based on these studies. In our study, we excluded malnourished patients and patients with low BMIs since we aimed to investigate the effect of surgery on postoperative nutritional status and on prognosis. Our study was structured based on these data, and reduced complication rates and shortened tube drainage times were observed after the preoperative nutrition program. The importance of pre-operative nutrition is emphasized in our study. However, one should note that the effect of an adequate and timely post-operative nutrition on prognosis is unquestionable. In a specific study conducted on this topic, early-stage nutrition within the scope of fast-track rehabilitation program resulted in lesser complications during the post-operative period and these patients were discharged sooner [15].

As with many cancer types, cachexia and low protein-albumin levels may be observed in lung cancer. A multifactorial process, including increased proinflammatory cytokines, reduced synthesis, increased cleavage, leakage into extravascular space, hypermetabolism and reduced protein intake, is involved in its mechanism [1, 3, 4, 16]. Serum albumin levels are dependent on the rate of synthesis, amount released from the liver cells, extent of distribution and breakdown in the bodily fluids. The daily loss of secreted albumin is 4 %. However, various pathological conditions may impact albumin metabolism. Reduction in synthesis secondary to hepatocyte damage, deficiency in amino acid intake, diseases involving acute or chronic inflammation may result in increased loss. In addition, serum albumin levels are also reduced in case of protein malnutrition, nephrotic syndrome, protein-losing enteropathy, burn, constructive pericarditis, ataxia telangiectasia and tumor-associated mesenteric blockage and mucosal diseases such as inflammatory bowel disease and hemodilution [17]. As a result, in the case of deficiency of protein, the building block of the body, all body functions will slow down, even stop. Obviously, this triggers development of morbidity and mortality [4, 7, 9]. Although there are studies advocating that complications in malnourished patients are caused by reduced immunity – increased infections and weakness of respiratory muscles, [18] slowed protein metabolism causes a multifactorial effect. However, with respect to malnutrition, the association of hypoalbuminemia and poor prognosis should not be attributed only to malnutrition, and it should be borne in mind that hypoalbuminemia is not observed only in malnourished patients.

There are many studies reporting how albumin levels affect mortality and morbidity, cause significant increases in postoperative complications and organ dysfunctions and cause prolonged hospitalizations and increased re-hospitalizations in patients who underwent surgery [1–3, 8, 11, 19, 20]. There are publications demonstrating that serum albumin levels have considerable effects on prognosis not only in lung cancer but also in other organ cancers [3, 9, 20]. A study on oesophageal cancers reported that albumin levels play an important role on prognosis as a general view, and suggests that preoperative nutrition support should be optimized in such patients [20]. In the study by Antoun et al. [2] it was found that low serum albumin levels generally increase morbidity and constitute the most important factor especially in the development of major complications. In the study on a very large population of patients that examined the mortality and morbidity rates during the first 30 days after surgery, it was concluded that serum albumin level is the best indicator with respect to patient data [11]. The literature review by Gupta [3] also reports that the most effective factor in the prognosis of patients with cancer is serum albumin level. Another study that followed up patients who underwent pneumonectomy investigated the risk of development of bronchopleural fistula, and found no other risk factor than low preoperative serum albumin levels [19]. In a multi-center study on patients who underwent pneumonectomy, it was found that recent smoking and extended resection as well as malnutrition affect major complications and mortality [1]. In our study, reduction in albumin levels was about 25 % in the control group while it is considerably lower (about 14 %) in the nutrition group. It was found that this difference led to less complications and shorter tube drainage times.

We think that one of the most significant results of our study was the reduction of 25 % in the postoperative albumin levels of the non-malnourished patients. Now we can predict that a patient whose preoperative albumin level is 4 mg/dl will have a postoperative albumin level of 3 mg/dl and increased complications. So, according to the results of our study, in order to maintain the postoperative albumin level at 3.5 mg/dl (the lower limit) or above, the preoperative albumin level should be about 4.5 mg/dl or above. We therefore concluded that, preoperative nutritional support may be recommended for all patients with preoperative serum albumin levels of < 4.5 mg/dl.

## Conclusion

The results of our study showed that, even in non-malnourished patients, a loss of 25 % may be observed in albumin levels during early postoperative period. Preoperative nutrition reduced the albumin loss occurring during the post-operative period. In addition, lesser complications occurred following lung cancer operation, resulting in discharge of patients within a shorter period.
